# Supplementary material for: Increasing associative plasticity in temporo-occipital back-projections improves visual perception of emotions
Source: Nat Commun. 2023 Sep 22;14:5720. doi: 10.1038/s41467-023-41058-3 (PMC10517146; doi:10.1038/s41467-023-41058-3)
Supplement: Supplementary file 1 — Supplementary Information [file 41467_2023_41058_MOESM1_ESM.pdf]

Increasing associative plasticity in temporo-occipital back-projections improves visual perception of emotions from facial stimuli

Sara Borgomaneri, Marco Zanon, Paolo Di Luzio, Antonio Cataneo, Giorgio Arcara,

Vincenzo Romei, Marco Tamietto and Alessio Avenanti

Supplementary Information

This PDF file includes:

- **Supplementary Fig. 1.** Experiment 1: TMS-evoked activity in the targeted V1/V2 and pSTS sites.
- **Supplementary Fig. 2.** Experiments 1–5: Study approach and main findings.
- **Supplementary Fig. 3.** Experiments 2, 3, and 5: Temporal dynamics of ccPAS aftereffects.
- **Supplementary Table 1.** Experiment 2, 3 and 4: Baseline d' values.
- **Supplementary Table 2.** Experiment 2, 3 and 4: Baseline response bias (β) values.
- **Supplementary Table 3.** Experiment 2, 3 and 4: Baseline response time (RT) values.
- **Supplementary Table 4.** Experiments 2, 3, and 4: Behavioral performance following ccPAS.
- **Supplementary Table 5.** Experiment 5: Behavioral performance at baseline and following ccPAS.
- **Supplementary Table 6.** Experiment 5: ERP response to face stimuli at baseline and following ccPAS.
- **Supplementary references.**

Supplementary Fig. 1. Experiment 1: TMS-evoked activity in the targeted V1/V2 and pSTS sites.

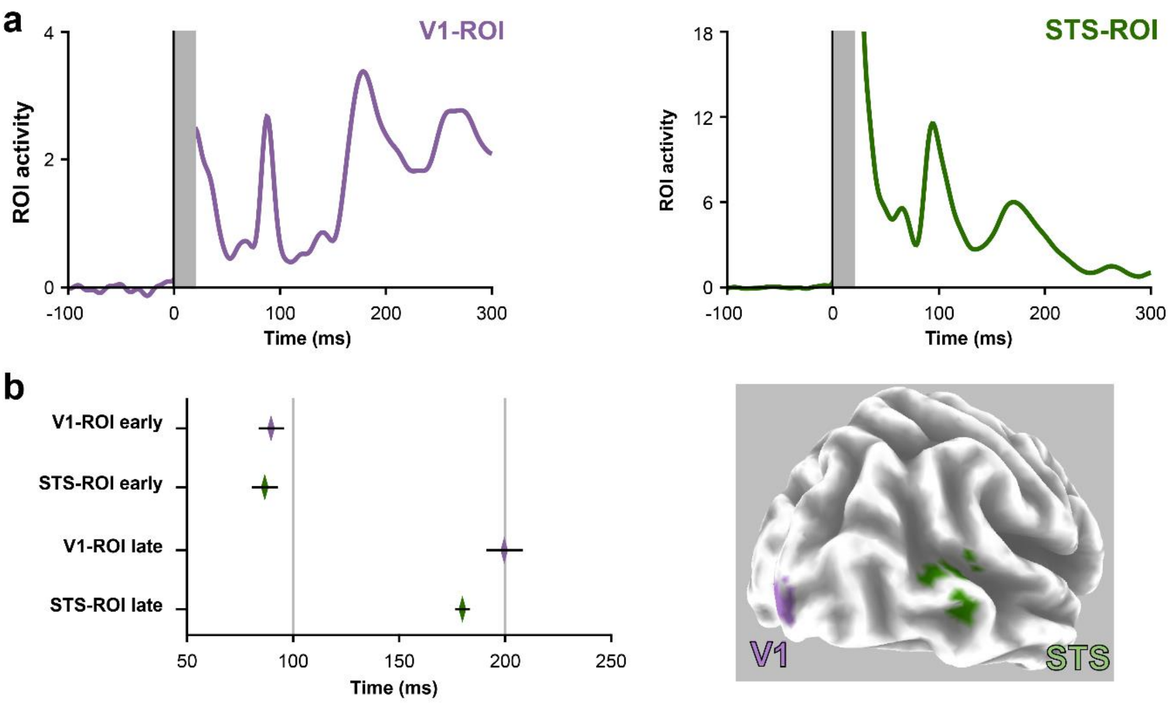

a TMS-evoked activity at the source level was extracted using sLORETA [S1, S2, S3]. We observed two peaks of activity in the targeted V1/V2 site (V1-ROI): a small and short-lasting peak occurring at ~90 ms from TMS, and a later peak with maximal amplitude occurring at ~200 ms. Similar activity patterns were detected in an ROI centered around the targeted pSTS site (STS-ROI). **b** The diamonds represent the mean latency ± S.E.M. of the two peaks evoked by TMS over the two ROIs. To assess whether the activation observed in V1/V2 was independent of that observed in pSTS and rule out volume conduction effects, we extracted peak latencies at the individual subject level and conducted a repeated-measures ANOVA with ROI (V1, STS) and Latency (90, 200) as within-subjects factors (N = 10 participants). The ANOVA revealed a significant main effect of Latency ($F_{1,9}$ = 521.08; p < 0.0001 η_p^2 = 0.98) and a significant Latency × ROI interaction ($F_{1,9}$ = 5.53; p = 0.043; η_p^2 = 0.38). Subsequent post-hoc analysis, corrected for multiple comparisons using the Duncan post-hoc test, demonstrated that mean latencies significantly differed between the two ROIs at about 200 ms (V1-ROI = 200 ms, 95% Confidence Interval (CI) [183, 216]; STS-ROI: 180, 95% CI [173, 187]; p = 0.004), thus indicating time-specific activation of V1/V2 following pSTS stimulation. In contrast, no significant latency difference between the ROIs was observed at 90 ms (V1-ROI = 90 ms, 95% CI [78, 102]; STS-ROI = 87 ms, 95% CI [75, 99]; p = 0.57). All statistical tests were two-tailed. Source data for latency results are provided as a Source Data file. Dataset to generate the EEG findings is provided at the following link: https://osf.io/yqbsj/

Supplementary Fig. 2. Experiments 1–5: Study approach and main findings.

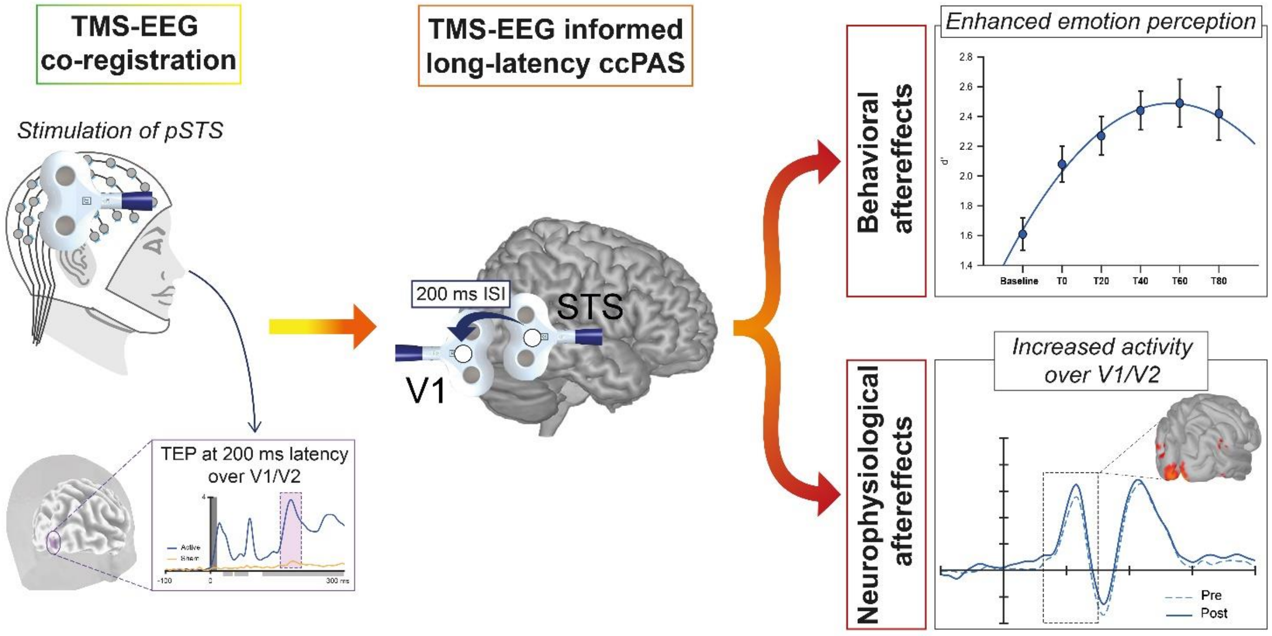

Schematic representation of the methodological approach and main findings. Using TMS-EEG co-registration, we estimated the timing of signal propagation from pSTS to V1/V2. Notably, we observed maximal activation of V1/V2 approximately 200 ms after pSTS stimulation. Leveraging this temporal information, we devised a long-latency (200-ms) ccPAS protocol aimed at enhancing synaptic efficacy within the pSTS-to-V1/V2 pathway. The critical ccPAS protocol involved the application of pairs of TMS pulses to stimulate the pSTS (first pulse) and V1/V2 (second pulse), inducing repeated and convergent activation of V1/V2 neurons, optimizing the induction of Hebbian associative plasticity. Following this protocol, the participants demonstrated enhanced visual perception of emotions from face stimuli, accompanied by increased temporo-occipital activations, particularly over the target site V1/V2 (i.e., the area of convergent activation during ccPAS). These compelling findings provide strong evidence supporting the crucial role of the pSTS-to-V1/V2 pathway in the perception of emotions from facial stimuli. Moreover, the research demonstrates the functional malleability of this pathway through the manipulation of associative plasticity. The EEG cap image displayed on the left side of the figure is utilized under a Shutterstock.com license and has been adapted from marina_ua/Shutterstock.com. The brain illustrated at the center of the figure is based on a T1-weighted image from the Colin27 brain template implemented in MRIcron. Chris Rorden's MRIcron, all rights reserved. https://people.cas.sc.edu/rorden/mricron/install.html.

Supplementary Fig. 3. Experiments 2, 3, and 5: Temporal dynamics of ccPAS aftereffects.

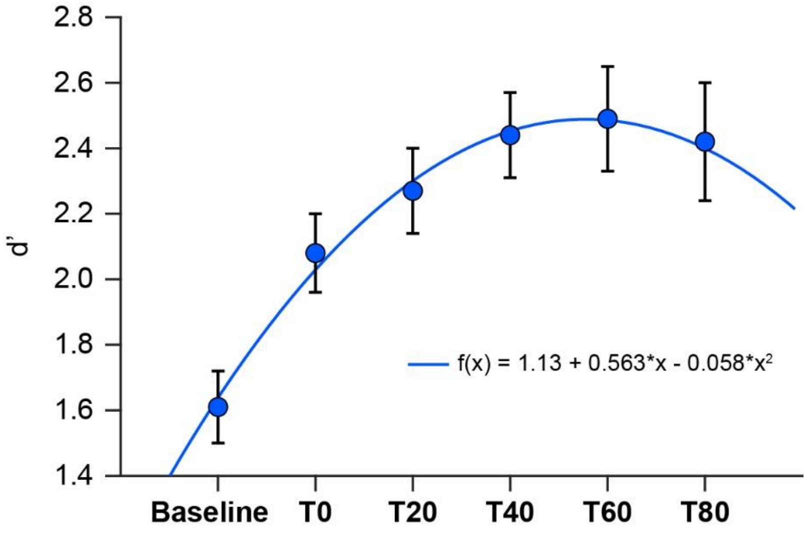

Changes in mean d' values before and after the critical ccPAS condition across Experiment 2, 3 and 5. We further examined the temporal unfolding of the ccPAS-induced increase in sensitivity to facial expressions in the 17-ms exposure condition by pooling together raw d' data from the three experimental groups (i.e., $Exp2_{STS\text{-}V1}$, $Exp3_{STS\text{-}V1}$, $Exp5_{STS\text{-}V1}$) and comparing d' values using a series of two-tailed *t*-tests. Compared to baseline (mean d' value = 1.61, 95% confidence intervals (CI) [1.39, 1.82]), a significant increment in emotion recognition performance was present throughout the 5 post-ccPAS sessions, specifically at T0 (d' = 2.08, 95% CI [1.85, 2.31]; $t_{44} = 5.90$; $p < 0.0001$; *Cohen's d* = 0.88; N = 45 participants), T20 (d' = 2.27, 95% CI [2.01, 2.52]; $t_{26} = 3.79$, $p = 0.0008$, *Cohen's d* = 0.73; N = 27 participants), T40 (d' = 2.44, 95% CI [2.18, 2.71]; $t_{26} = 4.80$, $p = 0.00006$, *Cohen's d* = 0.92; N = 27 participants), T60 (d' = 2.49, 95% CI [2.17, 2.80]; $t_{26} = 4.60$, $p = 0.0001$, *Cohen's d* = 0.89; N = 27 participants) and T80 (d' = 2.42, 95% CI [2.06, 2.77]; $t_{26} = 4.60$, $p = 0.0001$, *Cohen's d* = 0.88; N = 27 participants). All comparisons survived Bonferroni correction ($p = 0.05/15 = 0.003$). No statistical differences between post-ccPAS sessions were found (all $t \leq 1.68$, all $p \geq 0.10$). This suggests prolonged effects of the ccPAS protocol targeting pSTS-toV1/V2 feedback connections for at least 80 minutes. Additionally, mean d' values over the 6 time bins (Baseline, T0, T20, T40, T60, T80) were accurately fitted to a quadratic regression function, defined by the equation $y = a + bx + cx^2$ ($y = 1.13 + 0.563*x - 0.058*x^2$; SSE = 0.0046; $R^2_{adj} = 0.99$). The fitting revealed peak performance enhancement 40-60 minutes post-ccPAS and a decay trend afterward. All statistical tests were two-tailed. Error bars denote S.E.M. Source data are provided as a Source Data file.

Supplementary Table 1. Experiment 2, 3 and 4: Baseline d' values.

Measure	Experiment	Protocol	17 ms	33 ms	50 ms
Baseline d' values	**Experiment 2** (N=42)	$Exp2_{STS\text{-}V1}$	1.8 ± 0.6	2.8 ± 0.9	3.0 ± 0.9
		$Ctrl_{0ms}$	2.0 ± 0.4	2.8 ± 0.9	3.1 ± 0.8
		$Ctrl_{100ms}$	2.0 ± 1.0	2.4 ± 1.1	2.8 ± 1.1
	**Experiment 3** (N=39)	$Exp3_{STS\text{-}V1}$	2.0 ± 0.8	2.9 ± 0.8	3.5 ± 0.9
		$Ctrl_{V1\text{-}STS}$	2.3 ± 0.5	2.9 ± 0.5	3.3 ± 0.6
		$Ctrl_{Sham}$	1.9 ± 0.4	2.7 ± 0.6	3.0 ± 0.9
	**Experiment 4** (N=28)	$Ctrl\text{-}Gender_{STS\text{-}V1}$	2.0 ± 0.7	2.5 ± 0.8	3.1 ± 0.7
		$Ctrl\text{-}Gender_{Sham}$	1.9 ± 0.5	2.6 ± 0.7	2.9 ± 0.7

Mean d' ± standard deviation at baseline in Experiments 2, 3, and 4. We conducted a series of analyses to examine whether there were any differences in d' values before administering ccPAS across different stimulus exposure time conditions. A ccPAS × Exposure time ANOVA was conducted across all ccPAS groups in Experiments 2-4 (N = 109 participants). The results indicated a significant main effect of Exposure time ($F_{2,202}$ = 172.85; p < 0.0001; η_p^2 = 0.63), demonstrating that performance was better when stimuli were presented for 50 ms compared to 33 ms (p < 0.0001; *Cohen's d* = 0.61), and performance was lower when stimuli were presented for 17 ms compared to 33 ms (p < 0.0001; *Cohen's d* = 1.21) and 50 ms time exposure (p = 0.00001; *Cohen's d* = 1.60), as shown by post-hoc analyses conducted using the Duncan test to correct for multiple comparisons. Importantly, there was no significant main effect of ccPAS ($F_{7,102}$ = 0.73; all p = 0.65; η_p^2 = 0.05) or interaction with ccPAS ($F_{14,202}$ = 1.26; p = 0.24; η_p^2 = 0.08), suggesting comparable d' values across the different groups before ccPAS administration. These findings were consistently observed in all individual experiments. In Experiment 2 (N = 42 participants), the ccPAS × Exposure time ANOVA revealed a significant main effect of Exposure time ($F_{2,87}$ = 58.11; p < 0.0001; η_p^2 = 0.59), with better performance when stimuli were presented for longer durations on the screen, and no main effect of ccPAS ($F_{2,39}$ = 0.31; p = 0.73; η_p^2 = 0.02) or interaction with ccPAS ($F_{4,78}$ = 1.77; p = 0.14; η_p^2 = 0.08). Similarly, in Experiment 3 (N = 39 participants), a significant main effect of Exposure time was observed ($F_{2,72}$ = 66.62; p < 0.0001; η_p^2 = 0.65), while there was no significant main effect of ccPAS ($F_{2,36}$ = 1.08; p = 0.35; η_p^2 = 0.06) or interaction with ccPAS ($F_{4,72}$ = 1.39; p = 0.24; η_p^2 = 0.07). In Experiment 4 (N = 28 participants), a significant main effect of Exposure time was found ($F_{2,52}$ = 49.81; p < 0.0001; η_p^2 = 0.66), but no significant main effect of ccPAS ($F_{1,26}$ = 0.10; p = 0.75; η_p^2 < 0.01) or interaction with ccPAS ($F_{2,52}$ = 0.94; p = 0.40; η_p^2 = 0.03). All statistical tests were two-tailed. In summary, these analyses consistently demonstrated better performance with longer exposure duration, and importantly, similar baseline d' values across all groups in Experiments 2-4. Source data are provided as a Source Data file.

Supplementary Table 2. Experiment 2, 3 and 4: Baseline response bias (β) values.

Measure	Experiment	Protocol	17 ms	33 ms	50 ms
**Baseline β values**	**Experiment 2** (N=42)	$Exp2_{STS\text{-}V1}$	–0.4 ± 0.9	–0.3 ± 0.7	0.0 ± 0.9
		$Ctrl_{0ms}$	–0.2 ± 0.6	–0.1 ± 0.9	0.1 ± 0.8
		$Ctrl_{100ms}$	–0.0 ± 0.6	0.3 ± 1.0	0.3 ± 0.9
	**Experiment 3** (N=39)	$Exp3_{STS\text{-}V1}$	0.0 ± 0.7	0.0 ± 0.8	0.1 ± 0.9
		$Ctrl_{V1\text{-}STS}$	0.2 ± 0.7	0.3 ± 0.8	–0.0 ± 0.7
		$Ctrl_{Sham}$	0.1 ± 0.4	0.4 ± 0.8	0.4 ± 0.5
	**Experiment 4** (N=28)	$Ctrl\text{-}Gender_{STS\text{-}V1}$	-0.3 ± 0.4	0.1 ± 0.7	0.0 ± 0.8
		$Ctrl\text{-}Gender_{Sham}$	-0.3 ± 0.6	0.1 ± 0.9	0.0 ± 0.9

Mean β ± standard deviation at baseline in Experiments 2, 3, and 4. We conducted a series of analyses to examine whether there were any differences in β before administering ccPAS across different stimulus exposure time conditions. A ccPAS × Exposure time ANOVA showed no significant main effects or interactions either in Experiment 2 (N = 42 participants; all $F \leq 2.39$, all $p \geq 0.10$) or Experiment 3 (N = 39 participants; all $F \leq 0.78$, all $p \geq 0.48$), suggesting comparable response bias across groups performing the emotion perception task. In Experiment 4 (gender perception task; N = 28 participants) the same ANOVA revealed a significant main effect of Exposure time ($F_{2,52} = 4.08$; $p = 0.023$; $\eta_p^2 = 0.14$), suggesting a tendency to respond "male" to a greater extent with 17-ms exposure times compared to 33-ms ($p = 0.011$; *Cohen's d* = 0.65) and 50-ms exposure times (55-ms: $p = 0.045$; *Cohen's d* = 0.38), which in turn did not differ from one another (p = 0.49), as shown by Duncan tests. No main effect of ($F_{1,26} = 0.01$; $p = 0.93$; $\eta_p^2 < 0.01$) or interaction with the factor ccPAS were found ($F_{2,52} = 0.04$; $p = 0.96$; $\eta_p^2 < 0.01$). Moreover, the ANOVA across all ccPAS groups in Experiments 2-4 (N = 109 participants) confirmed there was no significant main effect of ccPAS ($F_{7,102} = 1.27$; $p = 0.27$; $\eta_p^2 = 0.08$) or interaction Exposure time × ccPAS ($F_{14,202} = 0.62$; $p = 0.85$; $\eta_p^2 = 0.04$), suggesting an overall comparable response bias at baseline across all groups in Experiment 2-4. All statistical tests were two-tailed. In summary, these analyses consistently demonstrated slower responses with the most briefly presented stimuli, and importantly, similar baseline d' values across all groups in Experiments 2-4. Source data are provided as a Source Data file.

Supplementary Table 3. Experiment 2, 3 and 4: Baseline response time (RT) values.

Measure	Experiment	Protocol	17 ms	33 ms	50 ms
**Baseline RTs values**	**Experiment 2**	$Exp2_{STS\text{-}V1}$	514 ± 131	503 ± 114	510 ± 125
		$Ctrl_{0ms}$	513 ± 109	501 ± 122	492 ± 115
		$Ctrl_{100ms}$	524 ± 106	488 ± 92	495 ± 104
	**Experiment 3**	$Exp3_{STS\text{-}V1}$	531 ± 167	500 ± 143	521 ± 191
		$Ctrl_{V1\text{-}STS}$	551 ± 119	519 ± 92	526 ± 103
		$Ctrl_{Sham}$	533 ± 129	516 ± 133	509 ± 135
	**Experiment 4**	$Ctrl\text{-}Gender_{STS\text{-}V1}$	546 ± 113	520 ± 110	499 ± 97
		$Ctrl\text{-}Gender_{Sham}$	544 ± 99	531 ± 102	516 ± 99

Mean RTs ± standard deviation at baseline in Experiments 2, 3, and 4. We conducted a series of analyses to examine whether there were any differences in RTs before administering ccPAS across different stimulus exposure time conditions. The analysis involved conducting a ccPAS × Exposure time ANOVA across all ccPAS groups in Experiments 2-4 (N = 109 participants). The results revealed a significant main effect of Exposure time ($F_{1.6,157.8} = 17.61$; $p < 0.0001$; $\eta_p^2 = 0.15$), with longer RTs when participants responded to stimuli presented for 17 ms compared to 33 ms ($p < 0.0001$; *Cohen's d* = 0.63) and 50 ms ($p = 0.0001$; *Cohen's d* = 0.42), whereas RTs were comparable for stimuli presented for 33 and 50 ms ($p = 0.78$), as shown by Duncan post-hoc tests. The ANOVA did not show a significant main effect of ccPAS ($F_{7,102} = 0.13$; all $p \geq 0.99$; $\eta_p^2 < 0.01$) or interaction with ccPAS ($F_{10.9,157.8} = 0.97$; $p = 0.47$; $\eta_p^2 = 0.06$). Further analysis revealed main effects of Exposure time in Experiment 2 ($F_{1.6,63.2} = 11.78$, p < 0.0001; $\eta_p^2 = 0.23$), Experiment 3 ($F_{1.4,50.0} = 3.49$, p = 0.054; $\eta_p^2 = 0.09$), and Experiment 4 ($F_{2,52} = 15.62$, p < 0.0001; $\eta_p^2 = 0.38$), confirming slower RTs for stimuli presented for 17 ms. However, there were no main effects of ccPAS in Experiment 2 ($F_{2.39} = 0.02$; $p = 0.98$; $\eta_p^2 < 0.01$), Experiment 3 ($F_{2.36} = 0.05$; $p = 0.95$; $\eta_p^2 < 0.01$) or Experiment 4 ($F_{1,26} = 0.05$; $p = 0.82$; $\eta_p^2 < 0.01$). Additionally, there were no significant interaction with ccPAS in Experiment 2 ($F_{3.2,63.2} = 2.37$; $p = 0.075$; $\eta_p^2 = 0.11$), Experiment 3 ($F_{2.8,50.0} = 0.36$; $p = 0.77$; $\eta_p^2 = 0.02$) or Experiment 4 ($F_{2,52} = 0.95$; $p = 0.39$; $\eta_p^2 = 0.04$). All statistical tests were two-tailed. In summary, these analyses consistently demonstrated worst performance with shorter exposure duration, and importantly, similar baseline RT values across all groups in Experiments 2-4. Source data are provided as a Source Data file.

Supplementary Table 4. Experiments 2, 3, and 4: Behavioral performance following ccPAS.

Experiment	Measure	Protocol	17 ms	33 ms	50 ms
Experiment 2	d’	Exp2$_{\text{STS-V1}}$	1.3 ± 0.3	1.1 ± 0.3	1.2 ± 0.3
		Ctrl$_{\text{0ms}}$	1.1 ± 0.3	1.0 ± 0.3	1.0 ± 0.3
		Ctrl$_{\text{100ms}}$	0.9 ± 0.5	1.1 ± 0.4	0.9 ± 0.3
	β	Exp2$_{\text{STS-V1}}$	0.1 ± 0.7	–0.0 ± 0.7	–0.3 ± 0.8
		Ctrl$_{\text{0ms}}$	–0.1 ± 0.6	–0.3 ± 0.9	–0.3 ± 0.8
		Ctrl$_{\text{100ms}}$	–0.1 ± 0.4	–0.4 ± 1.1	–0.4 ± 0.7
	RTs	Exp2$_{\text{STS-V1}}$	0.9 ± 0.1	0.9 ± 0.1	0.9 ± 0.1
		Ctrl$_{\text{0ms}}$	0.8 ± 0.2	0.8 ± 0.2	0.8 ± 0.2
		Ctrl$_{\text{100ms}}$	0.9 ± 0.2	0.9 ± 0.2	0.9 ± 0.1
Experiment 3	d’	Exp3$_{\text{STS-V1}}$	1.3 ± 0.3	1.1 ± 0.2	0.9 ± 0.1
		Ctrl$_{\text{V1-STS}}$	1.1 ± 0.2	1.0 ± 0.2	1.0 ± 0.2
		Ctrl$_{\text{Sham}}$	1.0 ± 0.2	1.1 ± 0.2	1.1 ± 0.3
	β	Exp3$_{\text{STS-V1}}$	–0.3 ± 0.7	–0.1 ± 0.6	–0.3 ± 0.9
		Ctrl$_{\text{V1-STS}}$	–0.3 ± 1.2	–0.4 ± 0.8	0.0 ± 0.7
		Ctrl$_{\text{Sham}}$	–0.2 ± 0.6	–0.1 ± 0.7	–0.1 ± 0.6
	RTs	Exp3$_{\text{STS-V1}}$	0.9 ± 0.2	1.0 ± 0.2	0.9 ± 0.3
		Ctrl$_{\text{V1-STS}}$	0.9 ± 0.1	0.9 ± 0.1	0.8 ± 0.1
		Ctrl$_{\text{Sham}}$	0.8 ± 0.1	0.9 ± 0.1	0.9 ± 0.1
Experiment 4	d’	Ctrl-Gender$_{\text{STS-V1}}$	1.0 ± 0.2	1.1 ± 0.2	0.9 ± 0.2
		Ctrl-Gender$_{\text{Sham}}$	1.2 ± 0.3	1.1 ± 0.3	1.1 ± 0.2
	β	Ctrl-Gender$_{\text{STS-V1}}$	–0.4 ± 0.5	–0.8 ± 0.8	–0.5 ± 0.7
		Ctrl-Gender$_{\text{Sham}}$	–0.4 ± 0.9	–0.8 ± 0.9	–0.4 ± 1.0
	RTs	Ctrl-Gender$_{\text{STS-V1}}$	0.8 ± 0.1	0.8 ± 0.1	0.8 ± 0.1
		Ctrl-Gender$_{\text{Sham}}$	0.8 ± 0.1	0.8 ± 0.1	0.8 ± 0.1

Mean baseline-corrected values (± standard deviation) of d prime (d’), response bias (β) and response time (RTs) as a function of ccPAS protocol and exposure time in Experiments 2, 3, and 4. In Experiments 2 and 3, the increased sensitivity (d’) to briefly presented emotional faces (Fig. 3 and Fig. 4 in the main text) was not due to changes in decision criteria or speed/accuracy trade-offs, as the ccPAS × Exposure time × Time from ccPAS ANOVAs on baseline-corrected β did not show any significant ccPAS × Exposure time interactions (Experiment 2: $F_{4,78} = 0.17$, $p = 0.95$, $\eta_p^2 = 0.01$; Experiment 3: $F_{4,72} = 0.73$, $p = 0.58$, $\eta_p^2 = 0.04$; Experiment 4: $F_{2,52} = 0.09$, $p = 0.98$, $\eta_p^2 = 0.001$), nor did the same ANOVA on RTs (Experiment 2: $F_{4,78} = 1.39$, $p = 0.24$, $\eta_p^2 = 0.07$; Experiment 3: $F_{4,72} = 0.47$, $p = 0.76$, $\eta_p^2 = 0.03$; Experiment 4: $F_{2,52} = 1.13$, $p = 0.33$, $\eta_p^2 = 0.04$). Moreover, no other significant effects were found in the analyses of β in Experiments 2-4 (Experiment 2: all $F \leq 1.96$, all $p \geq 0.15$; Experiment 3: all $F \leq 1.77$, all $p \geq 0.08$; Experiment 4: all $F \leq 2.49$, all $p \geq 0.09$, whereas in all experiments, the analysis of RTs showed a main effect of Time from ccPAS (Experiment 2: $F_{4,156} = 7.80$, $p < 0.0001$, $\eta_p^2 = 0.17$; Experiment 3: $F_{4,144} = 16.64$, $p < 0.0001$, $\eta_p^2 = 0.32$; Experiment 4: $F_{4,104} = 21.19$, $p < 0.0001$, $\eta_p^2 = 0.45$), revealing an effect of practice; RTs became progressively faster as time elapsed from ccPAS. Specifically, faster RTs were observed in T20-T40-T60-T80 blocks relative to T0 as shown by Duncan post-hoc tests (Experiment 2: all $p \leq 0.0008$; *all Cohen’s* $d \geq 0.49$; Experiment 3: all $p \leq 0.01$ *all Cohen’s* $d \geq 0.40$; Experiment 4: all $p \leq 0.004$; *all Cohen’s* $d \geq 0.54$). All statistical tests were two-tailed. Source data are provided as a Source Data file.

Supplementary Table 5. Experiment 5: Behavioral performance at baseline and following ccPAS.

	Measure	Protocol	Baseline values	Baseline-corrected T0 values
**Experiment 5 Behavioral measures**	**d'**	$Exp5_{STS\text{-}V1}$	1.1 ± 0.5	1.7 ± 0.6
		$Ctrl_{V1\text{-}STS}$	1.2 ± 0.4	1.1 ± 0.6
	**β**	$Exp5_{STS\text{-}V1}$	0.1 ± 0.4	0.4 ± 0.9
		$Ctrl_{V1\text{-}STS}$	0.3 ± 0.5	0.2 ± 0.3
	**RTs**	$Exp5_{STS\text{-}V1}$	694 ± 89	1.0 ± 0.1
		$Ctrl_{V1\text{-}STS}$	669 ± 62	0.9 ± 0.2

Mean values ± standard deviation of d', β and RT indices are presented at baseline (raw values) and T0 (baseline-corrected values). A series of 1-way ANOVAs with the factor ccPAS ($Exp5_{STS\text{-}V1}$, $Ctrl_{V1\text{-}STS}$) showed comparable performance between groups before ccPAS administration on d' ($F_{1,33} = 0.01$, $p = 0.93$), β ($F_{1,33} = 1.10$, $p = 0.30$) and RT ($F_{1,33} = 0.92$, $p = 0.34$) indices at baseline, although in this experiment, participants were much slower and slightly less accurate relative to Experiments 2-4, due to the different experimental procedures; these included the use of smaller visual stimuli to minimize saccades during EEG acquisition, instructions to minimize head and body movements to reduce EEG artifacts and the use of a single exposure time of 17 ms. Nevertheless, the ANOVA with the factor ccPAS ($Exp5_{STS\text{-}V1}$, $Ctrl_{V1\text{-}STS}$) conducted on baseline-corrected performance at T0 showed significant between-groups differences in d' (see Figure 6C in the main text), replicating the increase in sensitivity observed in Experiments 2 and 3; moreover, similar ANOVAs showed no differences on β ($F_{1,33} = 0.72$, $p = 0.40$) or RTs ($F_{1,33} = 3.52$, $p = 0.07$), suggesting that the increased sensitivity to emotional faces was not due to changes in decision criteria or speed/accuracy trade-offs. All statistical tests were two-tailed. Source data are provided as a Source Data file.

Supplementary Table 6. Experiment 5: ERP response to face stimuli at baseline and following ccPAS.

	Measure	Protocol	Baseline values	Baseline-corrected T0 values
**Experiment 5 ERPs left cluster**	**P1**	$Exp5_{STS\text{-}V1}$	6.9 ± 5.4	-0.3 ± 1.5
		$Ctrl_{V1\text{-}STS}$	5.2 ± 2.6	0.1 ± 1.0
	**N170**	$Exp5_{STS\text{-}V1}$	-3.2 ± 4.1	0.5 ± 2.4
		$Ctrl_{V1\text{-}STS}$	-1.9 ± 3.0	0.7 ± 1.5
	**P2**	$Exp5_{STS\text{-}V1}$	4.1 ± 4.1	0.0 ± 1.4
		$Ctrl_{V1\text{-}STS}$	5.0 ± 3.7	-0.2 ± 1.1
**Experiment 5 ERPs right cluster**	**P1**	$Exp5_{STS\text{-}V1}$	7.1 ± 3.3	1.3 ± 1.4
		$Ctrl_{V1\text{-}STS}$	5.9 ± 4.1	0.3 ± 1.5
	**N170**	$Exp5_{STS\text{-}V1}$	-2.9 ± 3.2	1.0 ± 1.0
		$Ctrl_{V1\text{-}STS}$	-2.3 ± 3.3	0.9 ± 2.0
	**P2**	$Exp5_{STS\text{-}V1}$	7.0 ± 4.3	0.1 ± 1.6
		$Ctrl_{V1\text{-}STS}$	7.3 ± 4.3	0.2 ± 1.6

Mean values (microvolt) ± standard deviation of P1, N170, and P2 amplitudes are presented at baseline (raw values) and T0 (baseline-corrected values). We conducted a series of ccPAS ($Exp5_{STS\text{-}V1}$, $Ctrl_{V1\text{-}STS}$) × Electrode cluster (left, right) ANOVAs on the three ERP components at baseline. The results showed no main effects of the factor ccPAS or interactions with the factor Electrode cluster across the P1 (all $F \leq 1.53$, all $p \geq 0.22$), the N170 (all $F \leq 0.98$, all $p \geq 0.33$) and the P2 components (all $F \leq 0.24$, all $p \geq 0.63$). The analysis showed more prominent P2 amplitudes in the right cluster than the left cluster ($F_{1,34} = 19.26$, $p < 0.001$; $\eta_p^2 = 0.36$), whereas the two clusters showed comparable P1 ($F_{1,34} = 0.63$, $p = 0.43$) and N170 amplitudes ($F_{1,34} = 0.01$, $p = 0.90$). These findings indicate similar electrophysiological responses to emotional faces before ccPAS administration. Subsequent ccPAS ($Exp5_{STS\text{-}V1}$, $Ctrl_{V1\text{-}STS}$) × Electrode cluster (left, right) ANOVAs on baseline-corrected ERP components only revealed an increase in P1 amplitudes over the right cluster in the $Exp5_{STS\text{-}V1}$ group (Fig. 6 in the main text). ANOVAs on the N170 and P2 components showed no significant effects (N170: all $F \leq 1.20$, all $p \geq 0.28$; P2: all $F \leq 0.84$, all $p \geq 0.36$). All statistical tests were two-tailed. Source data are provided as a Source Data file. Dataset to generate the EEG findings is available at the following link: https://osf.io/yqbsj/

Supplementary References

[S1] Pascual-Marqui, R. D. Standardized low resolution brain electromagnetic tomography (sLORETA): technical details Running title: sLORETA. Methods Find. Exp. Clin. Pharmacol. (2002).

[S2] Fuchs, M., Kastner, J., Wagner, M., Hawes, S., Ebersole, J. S. A standardized boundary element method volume conductor model. Clin. Neurophysiol. 2002 113, 702–712 (2002).

[S3] Jurcak, V., Tsuzuki, D., Dan, I. 10/20, 10/10, and 10/5 systems revisited: their validity as relative head-surface-based positioning systems. Neuroimage 34, 1600–1611 (2007).
