# Supplementary material for: Increasing associative plasticity in temporo-occipital back-projections improves visual perception of emotions
Source: Nat Commun. 2023 Sep 22;14:5720. doi: 10.1038/s41467-023-41058-3 (PMC10517146; doi:10.1038/s41467-023-41058-3)
Supplement: Supplementary file 3 — Reporting Summary [file 41467_2023_41058_MOESM3_ESM.pdf]

nature portfolio

Corresponding author(s): Sara Borgomaneri, Marco Tamietto, and Alessio Avenanti

Last updated by author(s): Jul 23, 2023

Reporting Summary

Nature Portfolio wishes to improve the reproducibility of the work that we publish. This form provides structure for consistency and transparency in reporting. For further information on Nature Portfolio policies, see our Editorial Policies and the Editorial Policy Checklist.

Statistics

For all statistical analyses, confirm that the following items are present in the figure legend, table legend, main text, or Methods section.

n/a	Confirmed	
☐	☒	The exact sample size (*n*) for each experimental group/condition, given as a discrete number and unit of measurement
☐	☒	A statement on whether measurements were taken from distinct samples or whether the same sample was measured repeatedly
☐	☒	The statistical test(s) used AND whether they are one- or two-sided<br>*Only common tests should be described solely by name; describe more complex techniques in the Methods section.*
☒	☐	A description of all covariates tested
☐	☒	A description of any assumptions or corrections, such as tests of normality and adjustment for multiple comparisons
☐	☒	A full description of the statistical parameters including central tendency (e.g. means) or other basic estimates (e.g. regression coefficient) AND variation (e.g. standard deviation) or associated estimates of uncertainty (e.g. confidence intervals)
☐	☒	For null hypothesis testing, the test statistic (e.g. *F*, *t*, *r*) with confidence intervals, effect sizes, degrees of freedom and *P* value noted<br>*Give P values as exact values whenever suitable.*
☒	☐	For Bayesian analysis, information on the choice of priors and Markov chain Monte Carlo settings
☒	☐	For hierarchical and complex designs, identification of the appropriate level for tests and full reporting of outcomes
☐	☒	Estimates of effect sizes (e.g. Cohen's *d*, Pearson's *r*), indicating how they were calculated

Our web collection on statistics for biologists contains articles on many of the points above.

Software and code

Policy information about availability of computer code

Data collection	EEG data were collected using the BrainVision Recorder software by Brain Products GmbH. Behavioral data were collected via a personal computer running Psychtoolbox Matlab2011b by MathWorks Inc. Database of visual stimuli and custom Matlab scripts used for data collection are available at the Open Science Framework (https://doi.org/10.17605/OSF.IO/YQBSJ). The Talairach coordinates of the stimulated sites were collected using the SofTaxic Navigator System by Electro Medical Systems. MNI coordinates were converted into Talairach space using GingerALE 2.3.1. Schematic representations of the targeted sites were created using MRIcron.
Data analysis	G*Power 3 software was used to estimate the sample size. EEG data were analyzed using EEGLAB2022.1 toolbox, running in Matlab2020b (Mathworks inc.), LORETA-key software (v20171101), and Brainstorm (open and evolving source software). Statistical analyses of ERP amplitudes and behavioral data were conducted with Statistica v. 12 (StatSoft, Inc.)

For manuscripts utilizing custom algorithms or software that are central to the research but not yet described in published literature, software must be made available to editors and reviewers. We strongly encourage code deposition in a community repository (e.g. GitHub). See the Nature Portfolio guidelines for submitting code & software for further information.

Data

Policy information about availability of data

All manuscripts must include a data availability statement. This statement should provide the following information, where applicable:

- Accession codes, unique identifiers, or web links for publicly available datasets
- A description of any restrictions on data availability
- For clinical datasets or third party data, please ensure that the statement adheres to our policy

Original data are available at Open Science Framework, https://doi.org/10.17605/OSF.IO/YQBSJ. All data supporting the findings of this study are provided in the Source Data file. Source data are provided with this paper.

Research involving human participants, their data, or biological material

Policy information about studies with human participants or human data. See also policy information about sex, gender (identity/presentation), and sexual orientation and race, ethnicity and racism.

Reporting on sex and gender	Sex at birth was assessed using self-reports and reported for each participant sample.<br>Sex was considered only to have balanced samples of female and male participants across experiments<br>Sex differences are not hypothesized for the current research question. We performed preliminary analyses including the factor sex but found no influence of this factor on behavioral or electrophysiological data. These additional analyses are not included in the article. However, information about the participants' sex at the individual level is available in the Source data file and in the data stored on the Open Science Framework, accessible through this DOI: https://doi.org/10.17605/OSF.IO/YQBSJ.
Reporting on race, ethnicity, or other socially relevant groupings	We collected sociodemographic information (sex, age) for each participant sample, but did include socially constructed or socially relevant categorization variables in our design. All participants were white University students residing in Italy.
Population characteristics	We recruited 155 healthy human participants, randomly assigned to different experiments/experimental groups, as follows:<br>Experiment 1: 10 participants (6 females and 4 males) with a mean age (±SD) of 22.1 years (±2.2).<br>Experiment 2: 42 participants (22 females and 20 males) with a mean age of 23.9 years (±2.2).<br>Experiment 3: 32 participants (15 females and 17 males) with a mean age of 23.6 years (±2.8).<br>Experiment 4: 28 participants (19 females and 9 males) with a mean age of 22.8 years (±2.5).<br>Experiment 5: 36 participants (15 females and 21 males) with a mean age of 22.9 years (±2.6).<br>All participants were right-handed and had normal or corrected-to-normal visual acuity in both eyes. None of the participants had any neurological, psychiatric, or medical problems, nor any contraindication to TMS. Information about the participants' age and sex/gender at the individual level is available in the Source data file and in the data stored on the Open Science Framework, accessible through this DOI: https://doi.org/10.17605/OSF.IO/YQBSJ.
Recruitment	Participants were recruited through a combination of printed and electronic advertisements displayed on notice boards at different University of Bologna sites, as well as through word of mouth.
Ethics oversight	Bioethics Committee at the University of Bologna

Note that full information on the approval of the study protocol must also be provided in the manuscript.

Field-specific reporting

Please select the one below that is the best fit for your research. If you are not sure, read the appropriate sections before making your selection.

☒ Life sciences ☐ Behavioural & social sciences ☐ Ecological, evolutionary & environmental sciences

For a reference copy of the document with all sections, see nature.com/documents/nr-reporting-summary-flat.pdf

Life sciences study design

All studies must disclose on these points even when the disclosure is negative.

Sample size	In Experiment 1 (TMS-EEG coregistration), we could not conduct a power analysis due to the lack of prior studies investigating the effect of STS stimulation on V1/V2. Therefore, we determined the sample size of 10 participants based on previous TMS-EEG coregistration studies that examined the visual system. These studies include Zanon et al. (2010, Brain Topogr, N=10), Thut et al. (2011, Curr Biol, N=8), Romei et al. (2012, Curr Biol, N=9), Koivisto et al. (2017, Neuropsychologia, N=12), Zazio et al. (2019, Brain Topogr, N=8), and also the recent work of Veniero et al. (2021, Nat Comm, N=11).<br>For Experiment 2-5 (ccPAS experiments) we considered that there is no straightforward way to perform a power analysis for complex designs with more than two factors and no prior CCPAS study tested pSTS-V1/V2 areas on emotion recognition. Therefore, we estimated the sample size for the experimental groups in Experiments 2 and 3 based on previous work conducted in our lab. These studies examined the effect of V5-V1 ccPAS on motion perception (Romei et al., 2016, Curr Biol; Chiappini et al., 2018, Curr Biol) and the effect of STS-rTMS on emotion perception (Paracampo et al., 2018, Neuropsychologia), all of which showed large effect sizes (mean Cohen's d = 1.10). Using G*Power 3

	software (Faul et al., 2007) with a power (1-Β) of 0.95 and an alpha level of 0.05, we estimated that a sample size of 11 participants would be sufficient to detect baseline vs. post-ccPAS differences in the experimental groups. To ensure robust results, we slightly increased the sample size to 13/14 participants for each experimental or control group in Experiments 2-4. Additionally, we increased the sample size to 18 participants for each group in Experiment 5, as we not only tested behavioral data but also collected physiological data. The resulting sample sizes for all the experiments were as follows: N=42 in Experiment 2, N=32 in Experiment 3, N=28 in Experiment 4, and N=36 in Experiment 5. These sample sizes are similar to or greater than those used in previous STS-rTMS studies on emotion perception, such as Pitcher et al. (2014, J Neurosci, N=10 and N=12), Candidi et al. (2015, Cortex, N=16), Paracampo et al. (2018, Neuropsychologia, N=16), Sliwinska & Pitcher (2018, Neuroimage, N=30), Ferrari et al. (2018, Cogn Aff Behav Neurosci, N=36), and Pitcher et al. (2020, Cereb Cortex, N=14).
Data exclusions	A total of 4 participants were excluded in the initial phases of the Experiments 2-4 because of technical failures. In Exp1 and Exp5, trials with noisy EEG signals were excluded following standard procedures. In Exp2-4, response times (RTS) were calculated by removing trials with an incorrect or slow (≥1 sec) response.
Replication	The main results of the study indicated that the experimental ccPAS protocol significantly improved the perception of emotions from facial stimuli presented briefly for 17 ms. These findings were consistently obtained in three independent experiments, specifically Experiment 2, 3, and 5.
Randomization	Participants were randomly allocated into experimental and control groups.
Blinding	In Experiment 1, there were no blinding procedures are there was only one group in which we assessed the latency of TMS-evoked response on EEG data. For behavioral data of Experiments 2-5, we implemented a double-blind procedure: participants were blinded to group allocation, and the experimenters who collected and analyzed the data were blinded to the ccPAS conditions. The experimenters who administered ccPAS were not blinded to group allocation because they had to set TMS parameters (i.e., order of pulses, ISI, and orientation of the coils).

Reporting for specific materials, systems and methods

We require information from authors about some types of materials, experimental systems and methods used in many studies. Here, indicate whether each material, system or method listed is relevant to your study. If you are not sure if a list item applies to your research, read the appropriate section before selecting a response.

Materials & experimental systems

n/a	Involved in the study
☒	☐ Antibodies
☒	☐ Eukaryotic cell lines
☒	☐ Palaeontology and archaeology
☒	☐ Animals and other organisms
☒	☐ Clinical data
☒	☐ Dual use research of concern
☒	☐ Plants

Methods

n/a	Involved in the study
☒	☐ ChIP-seq
☒	☐ Flow cytometry
☒	☐ MRI-based neuroimaging
